# Guideline of guidelines: a critical appraisal of the evidence for PSA retesting intervals

**DOI:** 10.1111/bju.16809

**Published:** 2025-07-03

**Authors:** Kiana K. Collins, Pradeep S. Virdee, Nia Roberts, Jason L. Oke, Brian D. Nicholson

**Affiliations:** ^1^ Nuffield Department of Primary Care Health Sciences University of Oxford Oxford UK; ^2^ Bodleian Health Care Libraries Oxford UK

**Keywords:** prostate‐specific antigen, prostate cancer, intervals, AGREE II, systematic review

## Abstract

**Objectives:**

To summarise the recommendations for prostate‐specific antigen (PSA) retesting intervals and to evaluate the evidence cited by each guideline by conducting a systematic review of clinical practice guidelines.

**Methods:**

We searched PubMed and the Turning Research into Practice (TRIP) database for guidelines written in English and developed or updated in 2013–2024. Guideline quality assessment was performed using the AGREE II tool. We narratively synthesised results.

**Results:**

Eleven guidelines were included. Ten (91%) recommended PSA retesting intervals of approximately 2 to 4 years. A total of 37 studies were referenced as evidence for the recommended intervals across the 11 guidelines. Five of these studies (14%) had the objective of determining PSA retesting intervals. Fourteen studies (38%) analysed single PSA test results. Five guideline recommendations partially aligned with the evidence referenced and five did not align.

**Conclusions:**

Generally, for asymptomatic patients aged ≥50 years with PSA levels between 1 and 3 ng/mL, most guidance recommended a retesting interval of 2–4 years, with the possibility to extend the interval to 4–10 years for patients with a PSA value <1 ng/mL. Until research generates direct evidence for PSA retesting intervals for both asymptomatic and symptomatic patients, clinicians and patients engaging in shared decision‐making should be aware that current guidelines lack direct evidence for recommended PSA retesting intervals.

**Abbreviations**
ACS: American Cancer Society
CPG: clinical practice guideline
ERSPC: European Randomised Study of Screening for Prostate Cancer
IQR: interquartile range
mpMRI: multiparametric MRI
NCCN: National Comprehensive Cancer Network
PLCO: Prostate, Lung, Colorectal, and Ovarian Cancer Screening Trial
USPSTF: US Preventive Services Task Force

## Introduction

Prostate cancer is the most commonly diagnosed cancer [1], and the second leading cause of cancer mortality in men in the United Kingdom [2]. Low‐risk prostate cancer may not progress in some patients, but the natural history of the disease is poorly understood [3].

Screening for prostate cancer with the PSA test remains controversial. Evidence from five randomised controlled trials of PSA screening [4] is inconsistent, particularly concerning its impact on prostate cancer mortality [5, 6, 7, 8, 9]. The European Randomised Study of Screening for Prostate Cancer (ERSPC) [5] reported a significant reduction in prostate cancer deaths among screened men, which increased to 51% after adjusting for control arm contamination and nonattendance over 13 years. Conversely, no significant mortality benefit was found in the Prostate, Lung, Colorectal, and Ovarian Cancer Screening Trial (PLCO) [6] or the Cluster Randomised Trial of PSA (CAP) [7]. However, both trials faced challenges with contamination where patients in the control arms opportunistically received PSA tests.

In the United Kingdom, both symptomatic [10] and asymptomatic patients [11] can request a PSA test, but there is no guidance for general pratition GPs about when or if to retest the patient if the PSA result falls below the threshold for referral to urology or secondary care. Retesting patients at the right evidence‐based intervals may preserve the benefit of early prostate cancer diagnosis while reducing the harms of overdiagnosis. The timing of PSA retesting intervals is entirely dependent on this trade‐off and no consensus exists on the optimal PSA retesting interval in primary care [12].

Clinical practice guidelines (CPGs) are essential tools for clinicians, providing evidence‐based frameworks to support their clinical decisions and optimise patient outcomes [13]. Due to updates from large randomised PSA screening trials, recommendations both for and against PSA screening change frequently. This occurred in 2018 when the US Preventive Services Task Force (UPSTF) published a guideline [14] that reversed its 2012 guidance advising against PSA screening. Changes in recommendations pose challenges by increasing uncertainty about PSA testing, for both GPs [15] and patients [16].

The aim of this review was to summarise recommendations for PSA retesting intervals in CPGs for symptomatic or asymptomatic patients in primary care. We examined the methods and outcomes of each study cited as evidence for the recommendations and determined if the recommendations were supported by appropriate evidence.

## Methods

### Evidence Acquisition

Following the Preferred Reporting Items for Systematic Reviews and Meta‐Analyses (PRISMA) [17] guidance, we conducted a systematic review of CPGs with recommended PSA retesting intervals in primary care for symptomatic or asymptomatic patients without a prior diagnosis of prostate cancer. Ethical approval was not required because we only included published articles. The study protocol was registered on the Open Science Framework (https://osf.io/k6whd).

### Search Strategies

We included national and international guidelines written in English. We searched PubMed, and adapted the search for the Turning Research into Practice (TRIP) [18] database and grey literature for the most up‐to‐date CPGs published between 2013 and 2024. The search strategy included terms such as: ‘detection’, ‘diagnosis’, ‘PSA’ or ‘screening.’ The full strategy is provided in Data S1.

### Selection Process

Two reviewers (K.K.C. and P.S.V.) independently conducted title and abstract screening and subsequently screened full‐text guidelines for eligibility using the screening tool Rayyan [19]. Any discrepancies were discussed until consensus. CPGs were eligible if they had a recommendation for PSA retesting intervals before the patient was diagnosed with prostate cancer. CPGs were included if they recommended PSA retesting intervals for asymptomatic or symptomatic patients. CPGs must have been produced with the support of a health professional association or government agency. Position, consensus or recommendation statements that were not fully endorsed by guideline committees were not included. CPGs were excluded if recommendations were for a cancer site other than prostate or recommended PSA retesting intervals for recurrence or active surveillance. CPGs were excluded if they did not recommend any PSA retesting intervals, recommended shared decision‐making only, or personalised retesting intervals as these recommendations were not specific intervals based on a number of years or months.

### Data Extraction

Data were extracted from eligible CPGs independently by two reviewers (K.K.C. and P.S.V.), with disagreements discussed until consensus. The following were extracted from the guidelines: guideline developer, year, country, recommended PSA retesting interval, references for interval recommendation, and information on whether symptoms were mentioned, whether retesting interval recommendations were stratified by risk (e.g. by age, PSA, ethnicity, family history, germline mutations), and when to stop retesting. The following information was extracted from the referenced studies within each guideline: author, year of publication, type of study, methods, whether single or multiple PSA tests were analysed, study outcomes, whether the study specifically aimed to calculate intervals and the PSA retesting interval suggestion.

### Data Synthesis

We conducted a narrative summary of CPGs that recommended PSA retesting intervals. We used the Oxford Centre for Evidence‐Based Medicine (OCEBM): Level of Evidence [20] to categorise the evidence. The categories included were: systematic review, randomised trial, model, prospective, retrospective, and guideline. We examined the research methods used in each study as well as the outcomes presented. To establish if the conclusions from the studies cited as evidence for retesting intervals were appropriately incorporated into CPG recommendations, we considered whether the recommendations aligned with the conclusion of the study. Alignment was categorised as ‘Yes’ if the CPG recommendation matched the referenced study. ‘Partial’ if the CPG recommended intervals that were similar to the study conclusions and ‘No’ if the CPGs referenced studies that recommended different intervals than the study recommended or cited studies that provided no explicit interval recommendation.

### Quality Assessment

The quality of each CPG was assessed using the AGREE II tool [21]. AGREE II includes 23 items across six domains: (1) scope and purpose; (2) stakeholder involvement; (3) rigour of development; (4) clarity and presentation; (5) applicability; and (6) editorial independence. K.K.C. and P.S.V. independently scored each item using a 7‐point scale (1‐strongly disagree to 7‐strongly agree). A standardised mean score for each of the seven domains was calculated using the formula: ([actual score—minimum score]/[maximum score—minimum score]) × 100% [21].

## Results

**Fig. 1:**
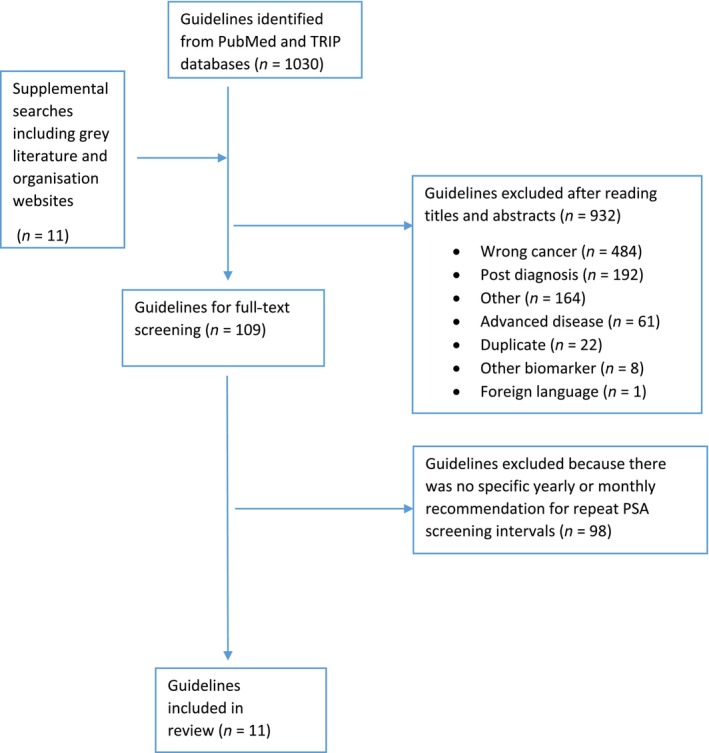
Preferred Reporting Items for Systematic Reviews and Meta‐Analyses (PRISMA) diagram. TRIP, Turning Research into Practice.

The initial search yielded 1030 CPGs, of which 11 were eligible for data extraction (Fig. 1). Across the 11 CPGs, 37 individual papers were referenced as evidence for the recommended PSA retesting intervals (Table S1). The most commonly cited paper [22] was cited in six (55%) of the CPGs [23, 24, 25, 26, 27, 28].

### Summary of CPG Recommendations for Repeat PSA Testing Intervals

Recommendations for the PSA retesting intervals ranged from 1 to 10 years (Table 1). Nine CPGs (82%) recommended an interval of approximately 2–4 years [23, 24, 25, 26, 27, 28, 29, 30, 31]. Ten (91%) of the interval recommendations were stratified by risk, but not the same type of risk [23, 24, 25, 26, 27, 28, 30, 31, 32, 33] (Table 1). Five (45%) were adjusted by both age and PSA value [23, 24, 25, 26, 33]. Three (27%) were adjusted by PSA value only [27, 30, 32]. Four (36%) recommended more frequent intervals based on risk factors such as family history, germline mutations (BRCA1 or BRCA2) and African ethnicity (Table 1) [26, 28, 31, 33]. Five (45%) recommended to start screening earlier (age 45 instead of age 50 years) for high‐risk patients but did not recommend shorter subsequent retesting intervals [23, 24, 27, 29, 30]. Five (45%) recommended to stop PSA testing at age 70 years [27, 29, 31, 32, 33] and six (55%) recommended to stop testing based on life expectancy and health status [23, 24, 27, 28, 30, 33]. PSA retesting intervals based on symptoms were not incorporated into any guidance.

**Table 1:** Clinical practice guideline findings.

Guideline	Country	Recommended PSA testing interval	References	Are the conclusions of the referenced studies aligned to the interval recommendation?*	Symptoms considered	Testing intervals stratified by patient risk	Recommendation on age to stop screening
American Cancer Society (2023) [30]	United States	2 years if PSA <2.5 ng/mL, 1 year if PSA ≥2.5 ng/mL	Wolf et al. [55], Smith et al. [56]	Yes	No	PSA <2.5 ng/mL and PSA ≥2.5 ng/mL	Stop testing patients with no symptoms and with <10 years life expectancy
American Urological Association (2023) [24]	United States	2–4 years	Vickers et al. [22], Carlsson et al. [39], Roobol et al. [71], Preston et al. [41], Vickers et al. [42], Heijnsdijk et al. [34], Gulati et al. [35], Heijnsdijk et al. [36], Ross et al. [37]	Partial	No	Re‐screening interval can be 1–4 years for patients with PSA levels of 1–3 ng/mL between the ages of 45–70 years. The re‐screening interval can be prolonged for patients aged 45–70 years with a PSA <1 ng/mL or those with a PSA below the age‐specific median. Possible to lengthen interval for patients PSA <1 ng/mL, age 60 years	Individual decision for when to stop based on life expectancy for patients aged 70–80 years. Can stop testing or substantially lengthen the re‐screening interval for patients aged ≥75 years if PSA is <3 ng/mL
Canadian Urological Association (2022) [27]	Canada	4 years if PSA <1 ng/mL, 2 years if PSA 1–3 ng/mL, more frequent if PSA >3 ng/mL	Vickers et al. [22], Gelfond et al. [40], Preston et al. [41]	No	No	PSA < 1 ng/mL, PSA 1–3 ng/mL, and PSA > 3 ng/mL	For men aged 60 years with a PSA < 1 ng/mL, consider discontinuing PSA screening. For all other men, discontinue PSA screening at age 70 years. For men with a life expectancy <10 years, discontinue PSA screening
Cancer Council Australia (2016) [29]^†^	Australia	2 years	Andriole et al. [6], Kilpeläinen et al. [72], Kjellman et al. [51], Bokhorst et al. [73], Hugosson et al. [74], Labrie et al. [8], Roobol et al. [75], Sandblom et al. [76], Andriole et al. [77], Sandblom et al. [78]	No	Mentions asymptomatic patients	No	Recommends to test men aged 50–69 years
EAU – EANM – ESTRO – ESUR – ISUP – SIOG (2024) [23]	Europe	Every 2 years for those initially at risk, or postponed up to 8 years in those not at risk	Vickers et al. [22], Carlsson et al. [39], Gelfond et al. [40], Roobol et al. [71]	Partial	Mentions both symptomatic and asymptomatic patients but does not recommend different retesting intervals based on symptom presentation	Follow‐up intervals of 2 years may be offered to those initially at risk (PSA >1 ng/mL at 40 years; PSA >2 ng/mL at 60 years)	Stop testing based on life expectancy and performance status. Patients with a life expectancy <15 years are unlikely to benefit from testing
French Urology Association Cancer Committee (2022) [28]	France	2–4 years but adapted to the patient's risk profile Annually patients with BRCA2 or HOXB13 germline mutations	Vickers et al. [22], Hugosson et al. [79], Preston et al. [41], Heijnsdijk et al. [34], Schröder et al. [5], Lilja et al. [43]	Partial	Mentions asymptomatic patients	Testing 2–4 years for patients aged >50 years, age 45 years for patients of Black ethnicity or with family history Start at age 40 years and test annually for those with BRCA2 or HOXB13	Stop testing patients with a life expectancy <10 years
Memorial Sloan Kettering (2016) [25]	United States	PSA ≥1 but <3 ng/mL: PSA testing every 2–4 years, PSA < 1 ng/mL: PSA testing at 6–10 years	Vickers et al. [22], Carlsson et al. [39], Andriole et al. [6], Schröder et al. [5], Thompson et al. [80], Lilja et al. [43], Loeb et al. [44], Eastham et al. [81], van Leeuwen et al. [45]	Partial	Mentions asymptomatic patients	Reported by age group (45–49, 50–59, 60–70 years) but generally the repeat testing interval was the same for each age group (2–4 years or 6–10 years depending on PSA)	Stop testing at age ≥76 years for all patients. Test patients if in good health 71–75 years. Stop testing if PSA ≤1 at age 60–70 years
NCCN (2023) [26]	United States	2–4 years for those with a PSA level ≤1 ng/mL, 1–2 years for high risk patients with PSA is ≤3 ng/mL and average risk patients with PSA 1‐3 ng/mL, and 1–2 years for those aged >75 years with PSA <4 ng/mL	Vickers et al. [22], Carlsson et al. [39], Roobol et al. [71], Preston et al. [41], Vickers et al. [42], Heijnsdijk et al. [34], Vertosick et al. [46], Preston et al. [47], Kovac et al. [48], Ulmert et al. [49]	Partial	No	High risk = Black/African American individuals, germline mutations that increase the risk for prostate cancer, and those with suspicious family history), repeat testing is recommended at 1–2 year intervals if PSA is ≤3 ng/mL	Stop testing at age 75 years unless patient is exceptionally healthy
Prostate Cancer Working Group and Ministry of Health (2015) [31]	New Zealand	2–4 years if PSA is in normal range and no family history. Annually if patient has family history	Basch et al. [82], Catalona et al. [83]	No	No	If patient has family history test every year if not 2–4 years. Same interval for all ages and PSA values	Patients aged over 70 years can be reassured further prostate cancer testing is not likely to be of any benefit
SEOM (2014) [32]	Spain	1–2 years if PSA <3 ng/mL, individualised risk assessment PSA 3–4, 6–12 months if PSA is >4 ng/mL	Andriole et al. [6], Schröder et al. [52]	No	No	PSA <3 ng/mL and PSA >4 ng/mL For PSA levels between 3 and 4 ng/mL, consider an individualised risk assessment that incorporates other risk factors. These factors include age, family history, ethnicity, DRE or PSA kinetics – no interval provided	Recommends to test men between ages of 50 and 70 years
South African Urology Association and the Prostate Cancer Foundation of South Africa (2024) [33]	South Africa	Age 45–49 years, PSA <1 ng/mL retest in 2 years, for patients PSA 1–2.5 ng/mL retest in 1 year. Age 50–59 years, PSA <1 ng/mL retest in 2 years, for PSA 1–3.5 ng/mL retest in 1 year. Age 60–70 years, PSA <1 ng/mL retest in 2 years, for PSA 1–4.5 ng/mL retest in 1 year	DeSantis et al. [84]	No	Mentions asymptomatic patients	Retesting intervals for patients of black ethnicity	Stop testing men aged >70 years or with a life expectancy <10 years

EANM, European Association of Nuclear Medicine; EAU, European Association of Urology; ESTRO, European Society for Radiotherapy and Oncology; ESUR, European Society of Urogenital Radiology; ISUP, International Society of Urological Pathology; SEOM, *Sociedad Espanola de Oncologia Medica*; SIOG, International Society of Geriatric Oncology.

* See Table S1 for PSA retesting interval recommendation evidence.

^†^ The Urological Society of Australia and New Zealand (2022) [85] published a position statement is to serve as an interim document for the optimised use of PSA testing in Australia and New Zealand until the Prostate Cancer Foundation of Australia and Royal Australian College of General Practitioners guidelines are updated. It recommends clinicians should follow the EAU position statement [66] risk stratified PSA retesting interval.

### Summary of Studies Referenced in CPGs as Evidence for PSA Retesting Intervals

**Fig. 2:**
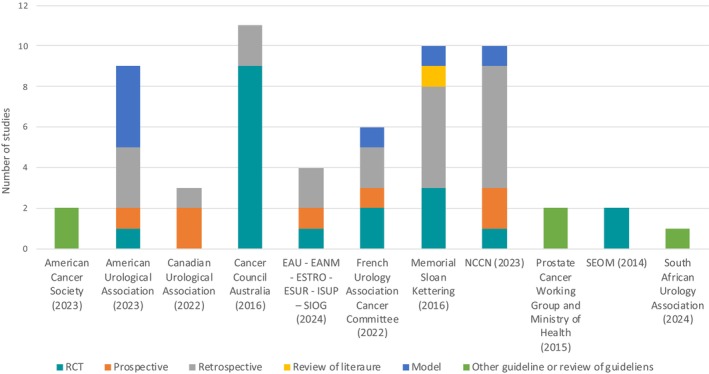
Referenced study evidence categorisation. EANM, Eurpoean Association of Nuclear Medicine; EAU, European Association of Urology; ESTRO, Euorpean Society for Radiotherapy and Oncology; ESUR, European Society of Urogenital Radiology; ISUP, International Society of Urological Pathology; NCCN, National Comprehensive Cancer Network; RCT, randomized controlled trial; SEOM, *Sociedad Espanola de Oncologia Medica*; SIOG, International Society of Geriatric Oncology.

A total of 37 studies were cited by CPGs. The most common study designs were randomised trials (41%) and retrospective studies (22%; Table S1), although the methods used in the randomised trial studies were more similar to retrospective cohort analyses. US and French CPGs [24, 25, 26, 28] referenced modelling studies (Fig. 2).

Five of the studies (14%), cited in two of the 11 CPGs [24, 26], specifically aimed to determine PSA retesting intervals [34, 35, 36, 37, 38] (Table S1). The remaining nine CPGs did not reference this type of applicable evidence [23, 25, 27, 28, 29, 30, 31, 32, 33]. Fourteen of the cited studies (38%) used a single baseline PSA test value to estimate the patients risk of prostate cancer diagnosis or mortality [22, 39, 40, 41, 42, 43, 44, 45, 46, 47, 48, 49, 50, 51] (Table S1). Seven of the 11 CPGs cited at least one of these studies as evidence for intervals [23, 24, 25, 26, 27, 28, 29].

The evidence cited by five of the CPGs did not align with the recommended PSA retesting interval reported in the cited studies [27, 29, 31, 32, 33] and five partially aligned with the studies cited [23, 24, 25, 26, 28] (Table 1). Recommendations did not align with the referenced studies for two reasons: (1) they either recommended different intervals than the study recommended [27], or (2) they cited studies which provided no explicit interval recommendation [29, 31, 32, 33] (Table S1).

### Quality of Guidelines

The AGREE II tool was used to appraise the overall quality of each guideline (Table 2). The scope and purpose (median score 72%, interquartile range [IQR] 69%–78%) and clarity of presentation domains (median score 69%, IQR 58%–90%) scored the highest overall. The range of scores in the editorial independence domain varied substantially, with two (18%) scoring below 10% [30, 33] and four (36%) scoring over 92% [23, 24, 25, 29]. The applicability domain had the second lowest overall score: a median of 42% with the least variability (IQR 34%–49%). The rigour of development domain had the lowest overall median score of 40% (IQR 34%–73%) but four CPGs [23, 24, 27, 29] scored over 70% in this domain. AGREE II scores by reviewer are provided Table S2.

**Table 2:** AGREE II appraisal scores.

Guideline	Domain 1: Scope and purpose	Domain 2: Stakeholder involvement	Domain 3: Rigour of development	Domain 4: Clarity of presentation	Domain 5: Applicability	Domain 6: Editorial independence	Overall scores
American Cancer Society [30]	69%	72%	31%	72%	46%	0%	48%
American Urological Association [24]	92%	86%	83%	64%	50%	92%	78%
Canadian Urological Association [27]	92%	56%	75%	81%	54%	50%	68%
Cancer Council Australia [29]	92%	89%	83%	83%	85%	96%	88%
EAU – EANM – ESTRO – ESUR – ISUP – SIOG [23]	56%	78%	69%	78%	42%	100%	71%
French Urological Association [28]	47%	28%	40%	75%	38%	63%	49%
Memorial Sloan Kettering [25]	89%	39%	40%	69%	31%	100%	61%
NCCN [26]	75%	67%	38%	72%	38%	79%	62%
Prostate Cancer Working Group and Ministry of Health [31]	61%	42%	38%	78%	48%	17%	47%
SEOM [32]	53%	28%	28%	69%	31%	50%	43%
South African Urology Association (2024) [78]	61%	33%	31%	58%	19%	8%	35%

EANM, European Association of Nuclear Medicine; EAU, European Association of Urology; ESTRO, European Society for Radiotherapy and Oncology; ESUR, European Society of Urogenital Radiology; ISUP, International Society of Urological Pathology; NCCN, National Comprehensive Cancer Network; RCT, randomized controlled trial; SEOM, Sociedad Espanola de Oncologia Medica; SIOG, International Society of Geriatric Oncology.

## Discussion

This review synthesised CPG recommendations for PSA retesting intervals. It examined the variation between CPG recommendations, assessed the quality of each CPG, and explored the applicability and methods of the studies cited as evidence for the PSA retesting interval recommendations.

We found recommended PSA retesting intervals varied widely. For a hypothetical patient of Black ethnicity, aged 55 years, with PSA level of 2 ng/mL, a test could be repeated annually based on the National Comprehensive Cancer Network (NCCN) or South African guidelines [26, 33], as long as 4 years based on the AUA guidelines [24] or 8 years according to the European Association of Urology [23]. This variation likely reflects the different intervals used in the randomised screening trials [4], ranging from annual screening in PLCO [6] to once every 7 years in the Belgian ERSPC cohort [52]. As a result, comparing the effect of different PSA retesting intervals on prostate cancer incidence and mortality remains unclear. Although these randomised trials have been instrumental in the domain of PSA screening, their primary objective was to ascertain whether screening conferred a mortality benefit, rather than to delineate the most suitable PSA retesting interval.

Most CPGs recommended PSA retesting intervals adjusted by risk, but stratification methods varied between age, PSA, and other risk factors. Risk stratification could be carried out for patients presenting with symptoms. A provincial guideline from British Columbia [53] recommended shorter intervals for patients presenting with symptoms but this was not supported by evidence on PSA retesting intervals for symptomatic patients. National Institute for Health and Care Excellence (NICE) NG12 guidance recommends to ‘consider PSA testing’ for patients presenting with LUTS, weight loss or erectile dysfunction, but lacks guidance on retesting with PSA if the patient does not qualify for referral.

In the absence of direct evidence for PSA retesting intervals [54], guideline developers rely on the best available evidence such as modelling, cohort studies and retrospective analyses of data from PSA screening trials. Cohort studies often focus on prostate cancer risk or mortality based on a single PSA measurement at a specific point in time [22, 39, 40, 41, 42, 43, 44, 45, 46, 47, 48, 49, 50, 51], rather than calculating retesting intervals. For instance, Preston et al. [41] concluded that risk of prostate cancer‐specific mortality in 30 years for patients with a baseline PSA below the median (0.68, 0.88, and 0.96 ng/mL for men aged 40–49, 50–54, and 55–59 years, respectively) was <2%. They argued for repeat testing at 5‐year intervals for patients aged 45 years with a baseline PSA level below the median. Studies reporting low PSA values as an indicator to defer or stop testing [39] are relevant to advocate for the extension of PSA retesting intervals but do not give evidence for the exact timing of the retesting intervals. Vickers et al. [22] suggested that one possible cut‐off point to determine more vs less frequent screening would be ≤1 ng/mL. They found that the risk of metastasis within 15 years was <0.4%, suggesting that a retesting interval of < 5 years was unnecessary for those patients. This type of research is crucial to understand the relationship between PSA values and the progression of prostate cancer but may not be suitable evidence to underpin recommendations for PSA retesting intervals.

Six modelling studies aimed to calculate retesting intervals and quantified the potential harms and benefits of reducing mortality with differing retesting intervals [34, 35, 36, 37, 38, 54]. The AUA [24] cited Gulati et al. [35], a microsimulation study of 35 different screening strategies. They found that a strategy that screens patients aged 50–74 years annually with a PSA threshold for biopsy referral of 4 ng/mL reduced the risk for prostate cancer death to 2.2%, with risk for overdiagnosis of 3.3%. Compared to a strategy that uses higher PSA thresholds for biopsy referral in older patients achieves a similar risk for prostate cancer death (2.2%) but reduced the risk for overdiagnosis to 2.3%. The study did not make a specific interval recommendations, but asserted that extending PSA retesting intervals might be acceptable for certain patients with low PSA levels.

When assessing the alignment of CPG recommendations with study conclusions the American Cancer Society (ACS) [30] was the only CPG where the recommendation matched the evidence cited. However, the ACS [30] exclusively referenced reviews and guidelines from its own organisation [55, 56]. These are not appropriate sources for evidence‐based recommendations designed for the general population. We found that 10/11 CPGs cited evidence without accurately reflecting their conclusions [23, 24, 25, 26, 27, 28, 29, 31, 32, 33]. This observation held true for various study types, including those focusing on interval calculations and those focused on risk of cancer mortality. In the modelling study by Heijnsdijk et al. [34], it was noted as safe to extend screening intervals beyond 2 years. Heijnsdijk et al. did not explicitly recommend 2‐to‐4‐year intervals, contrary to the recommendations provided by the AUA [24] and NCCN [26]. This type of misalignment underscores challenges in evidence‐based guideline development. We found no examples where the CPG recommendation aligned with the findings of primary research. However, the determination of PSA retesting intervals involves multiple complexities, including the type of screening (population‐based vs opportunistic), variations in prostate cancer prevalence, different PSA referral thresholds, the impact of accessibility and costs, and the role of additional tests such as pre‐biopsy MRI.

### Strengths and Limitations

This systematic review is the first to synthesise guideline recommendations for PSA retesting intervals and compare the studies cited as evidence with the guideline recommendations. The AGREE II tool was used to appraise overall guideline quality and we additionally looked at each study cited for the recommended PSA retesting interval. We evaluated which studies were designed with the aim of determining retesting intervals and whether these studies used single PSA test results or multiple PSA results to define intervals. We did not conduct a wider search of the literature to determine what could or should have been included in the CPG as each guideline committee has its own internal processes to select supporting evidence. Our focus was limited to CPGs recommending PSA retesting intervals to assess whether the evidence appropriately aligned with recommendations. As a result, we did not include CPGs that recommended shared decision‐making. For example, the USPSTF [14] recommended shared decision‐making when considering an initial PSA test but did not recommend any subsequent PSA retesting intervals. Other organisations, such as Prostate Cancer UK [12], the Prostate Cancer Risk Management Programme, the Japanese Urological Association [57] and the European Society for Medical Oncology [58, 59], recommend for PSA testing but provide no specific guidance on retesting intervals. We did not use the GRADE tool to assess the evidence because we were not appraising the bias or strength of the evidence. Instead, we aimed to assess the methods used in papers cited by CPGs to support their interval recommendations.

### Clinical Implications

The optimal PSA retesting interval is unknown, with no direct evidence or consensus on which guideline developers can base their recommendations. Despite this, many CPGs included in this review recommended a retesting interval of 2–4 years. Clinicians following these recommendations should take caution until direct evidence for PSA retesting intervals has been established.

Current guidance on PSA retesting is primarily focused on the outcome of prostate cancer mortality but this may not align with patient priorities. Patients weigh mortality benefits against risks such as unnecessary biopsies or urinary and bowel incontinence, valuing these trade‐offs differently [60]. This suggests a one‐size‐fits‐all repeat PSA testing approach may not reflect the preferences of patients [60].

### Policy Implications

Many CPGs recommend personalised PSA retesting intervals [14, 23, 28]. This flexibility aligns with the shared decision‐making approach, but it provides no clear guidance on determining appropriate intervals based on risk. This leaves room for varying interpretations from no testing to regular testing. Shared decision‐making may be the best solution to provide flexibility and account for the lack of consensus direct evidence for PSA retesting intervals. However, it remains unclear who should initiate the PSA discussion. If responsibility lies with the patient, education level and health literacy may lead to healthcare inequalities [61].

We found that no guidelines considered multiparametric MRI (mpMRI) results when determining PSA retesting intervals. Advances in mpMRI technology reduce clinically insignificant prostate cancer diagnoses by half [62], improve detection of significant cancers [63], and decrease unnecessary biopsies by 28% [64] to 48% [65]. With reduced overdiagnosis, more frequent PSA retesting intervals may become feasible. Future guideline updates should incorporate these advancements to improve PSA testing recommendations.

The UK National Screening Committee, Canadian Task Force on Preventative Health Care and the Danish Urological (Prostate) Cancer Group recommended against population‐wide PSA testing. As well as consensus on retesting intervals, international consensus should also be reached on whether or not to use PSA in asymptomatic patients.

### Research Implications

Risk‐adapted testing is a potential solution [66]. The European Union's Prostaforum 2022 declaration advocates for integrating new technologies into screening algorithms for population‐based screening programmes [67]. PRAISE‐ U is a European Union project aiming to align prostate cancer screening protocols and guidelines across member states by trialling a risk‐adapted PSA testing strategy [68]. Germany's PROBASE is a population‐based randomised trial exploring risk‐adapted PSA screening intervals. Low‐risk patients (PSA <1.5 ng/mL) are tested every 5 years, intermediate‐risk patients (PSA 1.5–2.9 ng/mL) are tested every 2 years but the screening interval is based on an initial midlife PSA test result. Results for prostate cancer‐specific incidence and mortality are not published yet. We argue for the importance of considering repeat PSA testing over time and adapting PSA retesting intervals accordingly. The role of repeat testing for cancer detection is currently under investigation in the Blood Test Trend for Cancer Detection study [69]. Appropriate evidence to support interval recommendations could be derived from carefully designed cohort studies, retrospective analyses of electronic medical records, modelling or machine learning studies. The outputs from these studies should then be trialled accordingly. Future research on PSA retesting intervals should include studies incorporating mpMRI scanners. The TRANSFORM Trial in the United Kingdom will start recruitment in 2025. It aims to compare potential screening options, including fast MRI scans trialled in PROSTOGRAM [63], genetic testing and PSA testing [70].

## Conclusion

Recommendations for PSA retesting intervals for asymptomatic patients before a prostate cancer diagnosis varied among guidelines without consensus on the optimal PSA retesting approach. No guidelines recommended PSA retesting intervals for patients presenting with symptoms of prostate cancer. To support the PSA retesting interval recommendations, most guidelines relied on indirect evidence derived from studies investigating a single PSA value, assessments of risk of prostate cancer progression, or data from randomised screening trials primarily aimed at mortality reduction rather than determining retesting intervals. Generally, for asymptomatic patients aged ≥50 years with PSA levels between 1 and 3 ng/mL, most guidance recommended a retesting interval of 2–4 years with the possibility to extend the interval to 4–10 years for asymptomatic patients with a PSA value <1 ng/mL. Until research generates direct evidence for PSA retesting intervals for both asymptomatic and symptomatic patients, clinicians and patients engaging in shared decision‐making should be aware that current guidelines lack direct evidence for recommended PSA retesting intervals.

## Funding

K.K.C. was funded for this work supported by Cancer Research UK (CRUK) grant number CANTAC721\100004. B.D.N. was funded by a CRUK Clinical Careers Committee Postdoctoral Fellowship (RCCPDF\100005). P.S.V. was funded by a National Institutes for Health Research School for Primary Care Research Postdoctoral Fellowship (C092).

## Disclosure of Interests

The authors declare that they have no competing interests.

## Supporting information


**Data S1.** Search strategy.
**Table S1**. Studies cited by guidelines as evidence for their recommended repeat PSA testing intervals.
**Table S2**. Individual AGREE II reviewer scores by guideline.
