# Supplementary material for: Guideline of guidelines: a critical appraisal of the evidence for PSA retesting intervals
Source: BJU Int. 2025 Jul 3;136(3):372–84. doi: 10.1111/bju.16809 (PMC12343985; doi:10.1111/bju.16809)
Supplement: Supplementary file 1 — Data S1. Search strategy. Table S1. Studies cited by guidelines as evidence for their recommended repeat PSA testing intervals. Table S2. Individual AGREE II reviewer scores by guideline. [file BJU-136-372-s001.docx]

## Supplementary Material

## Search strategy

**Search run January 2025**

**PubMed = 107**

(("prostate neoplasms"[Text Word] OR "prostatic neoplasms"[Text Word] OR "prostate cancer"[Text Word] OR "prostatic cancer"[Text Word] OR "prostate carcinoma"[Text Word] OR "prostatic carcinoma"[Text Word]) AND ("screening"[Text Word] OR "psa"[Text Word] OR "diagnosis"[Text Word] OR "detection"[Text Word])) AND (guideline[Filter] OR practiceguideline[Filter])

**Search for TRIP = 923**

("prostate neoplasms" OR "prostatic neoplasms" OR "prostate cancer" OR "prostatic cancer" OR "prostate carcinoma" OR "prostatic carcinoma") AND ("screening OR "psa" OR diagnosis OR detection) - limited to Guidelines within Secondary Evidence

Grey literature search: NICE, UPSTF, UK Screening Comittee, Canadian Preventitive Task Force, New Zealand Ministry, Danish ministry, Canadian Urological Association, American Urological Association, ESMO.

## Supplementary Table 1: Studies cited by guidelines as evidence for their recommended repeat PSA testing intervals

| **Paper** | **Referenced in Guidelines** | **Type of Study** | **Methods** | **Single or multiple test** | **Outcomes** | **Does the study specifically aim to calculate intervals and quantify risk for screening intervals** | **Interval recommendation** |
| --- | --- | --- | --- | --- | --- | --- | --- |
| Vickers (2013)[1] | EAU, AUA, Memorial Sloan, NCCN, CUA, France | Retrospective Case-control study | Single baseline PSA test can predict risk of prostate cancer | Single PSA test | Metastases, prostate specific mortality | No | PSA <1 testing more than every 5 years is unnecessary |
| Carlsson (2014)[2] | EAU, AUA, Memorial Sloan, NCCN | Retrospective cohort study | Baseline PSA to stratify prostate cancer risk based on cumulative hazard | Single PSA test | Prostate cancer diagnosis, metastasis, and death | No | PSA <1 at age 60 don’t need to test again based on 15 year risk |
| Gelfond (2015)[3] | EUA, CUA | Prospective cohort study | Kaplan-Meier and cox regressions predicting risk from baseline PSA test to prostate cancer diagnosis | Single PSA test | Prostate cancer diagnosis | No | PSA <1 10 years |
| Roobol (2005)[4] | EAU, AUA, NCCN | Retrospective cohort study based on ERSPC | Assessed PSA values and number of cancers detected | Single PSA test – but patient had multiple tests where they were <1 | Prostate cancer diagnosis | No | PSA <1 8 years |
| Preston (2016)[5] | AUA, NCCN, CUA, France | Prospective case control | Baseline PSA and risk of prostate cancer by age | Single PSA test | Lethal prostate cancer | No | PSA level below the median at age 45 years followed by repeat measurements at 5-year intervals. People with PSA <1.0 age 60 years are unlikely to develop lethal disease |
| Andriole (2012)[6] | Memorial Sloan, SEOM, Cancer Australia | Randomised trial PCLO | Relative risk of prostate cancer diagnosis and mortality | NA | Diagnosis and prostate cancer specific mortality | No | No interval recommendation. The study was an RCT with annual screening |
| Vickers (2010)[7] | AUA, NCCN | Retrospective case control | Multiple regression to find predict risk from PSA test at age 60 | Single PSA test | Diagnosis, metastasis or prostate cancer specific mortality | No | PSA<1 at age 60 it is safe to never test again |
| Heijnsdijk (2020)[8] | AUA, NCCN, France | Model | Microsimulation model assessing screening policies. Evaluated the following strategies: lengthening the screening interval when PSA was below 1.0 ng/mL at age 45 or 50 years, discontinuing screening when PSA was below 1.0 ng/mL at age 60 years, and biennial screening for all men | Multiple PSA tests over time using trajectories | Number of tests, overdiagnosis and lives saved | Yes. Compared with biennial screening for ages 45–69 years, lengthening screening intervals for men with PSA less than 1.0 ng/mL at age 45 years led to 46.8–47.0% fewer tests 0.9–2.1% fewer overdiagnoses, and 3.1–3.8% fewer lives saved. Stopping screening when PSA was less than 1.0 ng/mL at age 60 years and older led to 12.8–16.0% fewer tests, 5.0–24.0% fewer overdiagnoses, and 5.0–13.1% fewer lives saved | Can lengthen screening instead of test every 2 years**.** |
| Schroder (2014)[9] | Memorial Sloan, France | Randomised trial ERSPC | Rate ratio prostate cancer incidence and mortality | NA | Diagnosis and prostate cancer specific mortality | No | No interval recommendation - Different intervals ranging from 2-7 years |
| Schroder (2012)[10] | SEOM | Randomised trial ERSPC | Rate ratio prostate cancer incidence and mortality | NA | Diagnosis and prostate cancer specific mortality | No | No interval recommendation - Different intervals ranging 2-7 years |
| Gulati (2013)[11] | AUA | Model | Microsimulation model of 35 screening strategies that varied by start/stop ages, inter-screening intervals, and thresholds for biopsy referral. | Multiple PSA tests over time | Prostate cancer incidence and mortality | Yes. A reference strategy that screened men aged 50 to 74 years annually with a PSA threshold for biopsy referral of 4 µg/L reduced the risk for prostate cancer death to 2.15%, with risk for overdiagnosis of 3.3%. A strategy that used higher PSA thresholds for biopsy referral in older men achieved similar risk for prostate cancer death (2.23%) but reduced the risk for overdiagnosis to 2.3% | PSA screening strategies that use higher thresholds for biopsy referral for older men and that screen men with low PSA levels less frequently, can reduce harms while preserving lives saved compared to standard screening. |
| Heijnsdiik (2012) [12] | AUA | Model | Microsimulation Screening Analysis (MISCAN) to predict the number of prostate cancers, treatments, deaths and QALYs gained after the introduction of PSA screening | Multiple PSA tests over time. Screening strategies simulated included: annual screening in the age groups 55–69 years and 55–74 years, screening at 4-year intervals between 55–69, and single screens performed either at age 55, 60 or 65 years | Prostate cancer, treatments, deaths and QALYs | Yes. Per 1,000 men of all ages followed for their entire lifespan they predicted for annual screening from age 55–69 years: 9 fewer deaths due to prostate cancer (28% reduction), 14 fewer men receiving palliative therapy (35% reduction), and 73 life-years gained (average 8.4 years per prostate cancer death avoided). QALYs gained were 56 (range: −21, 97), a reduction of 23% from unadjusted life-years gained. The number needed to screen was 98 and number needed to detect 5. | Annual screening of all men between the ages of 55 and 74 resulted in more life-years gained but the same number of QALYs. |
| Ross (2000) [13] | AUA | Model | Monte-Carlo simulation based on a Markov model was used to simulate the natural history of prostate cancer using different starting ages, testing intervals, and PSA thresholds for prostate biopsy | Multiple psa tests over time. Tested biennial and annual testing | Numbers of prevented prostate cancer deaths, PSA tests, and prostate biopsies per 1000 men aged 40 through 80 years, compared among 7 different strategies vs no screening. | Yes. Annual screening strategy at age 50 prevented 3.2 deaths, with an additional 10,500 PSA tests and 600 prostate biopsies, while the less frequent strategy (2 year interval) prevented 3.3 deaths, with an additional 7500 PSA tests and 450 prostate biopsies. | Screening strategy at age 40 and 45 years with a 2-year testing interval after age 50 years may be both more effective and require less testing than the standard strategy of annual PSA testing beginning at age 50 years. |
| Thompson (2006) [14] | Memorial Sloan | Model | Logistic regression was used to model the risk of prostate cancer and high-grade disease associated with age at biopsy, race, family history of prostate cancer, PSA level, PSA velocity, DRE result, and previous prostate biopsy | Multiple PSA tests over time to compute PSA velocity | Prostate cancer diagnosis | No | Predictive model allows an individualised assessment of prostate cancer risk and risk of high-grade disease for men who undergo a prostate biopsy |
| Lilja (2007) [15] | Memorial Sloan, France | Retrospective case control | Logistic regression to estimate the risk of prostate cancer | Single test | Prostate cancer diagnosis | No | A single PSA test at age 44 to 50 years predicted prostate cancer. This raises the possibility of risk stratification for screening programs |
| Loeb (2012) [16] | Memorial Sloan | Review of literature | Review of baseline PSA testing at age ≤60 to predict prostate cancer risk and prognosis | Single test | Prostate cancer diagnosis | No | Baseline PSA measurements at a young age were significant predictors of later prostate cancer diagnosis and disease-specific outcomes. Baseline PSA testing may be used for risk stratification and to guide screening protocols. |
| Eastham (2003) [17] | Memorial Sloan | Retrospective cohort study | Analysis of an unscreened population of 972 men (median age, 62 years). Five consecutive blood samples were obtained during a 4-year period and were assessed for total and free PSA levels. | Multiple tests | How often a participant's PSA level would return to normal the year after the level had been elevated | No | Among men with an abnormal PSA, a high proportion had a normal PSA finding at 1 or more subsequent visits during 4-year follow-up: 68 (44%) of 154 participants with a PSA level higher than 4 ng/mL; 116 (40%) of 291 had a level higher than 2.5 ng/mL; 64 (55%) of 117 had an elevated level above the age-specific cutoff; and 76 (53%) of 143 had a level between 4 and 10 ng/mL and a free-to-total ratio of less than 0.25 ng/mL. |
| Ven Leeuwen (2010) [18] | Memorial Sloan | Retrospective cohort study – ERSPC  Secondary analysis of ERSPC | Age adjusted cumulative hazards | Single test | Prostate cancer incidence and mortality | No | Adjusted absolute difference in prostate cancer specific mortality between the intervention population and the clinical population increased with increasing PSA level at study entry |
| Vertosick (2020) [19] | NCCN | Retrospective cohort | Absolute risks of prostate cancer metastasis or death at 10, 15 and 20 years were calculated using Kaplan-Meier methods | Single test | Prostate cancer metastasis or mortality | No | Patients 60 years old with PSA below median (less than 1.2 ng/ml) had 0.4% risk of prostate cancer death at 20 years. Screening should focus on men in top PSA-quartile at age 60. Men with elevated PSA but a low 4Kscore can safely be monitored with repeated blood markers in place of immediate biopsy |
| Preston (2019) [20] | NCCN | Prospective case control | Logistic regression estimated odds ratios for prostate cancer by category of baseline PSA. | Single test | Prostate cancer incidence and aggressiveness | No | PSA levels in midlife strongly predicted total and aggressive prostate cancer in black men. Targeted screening based on a midlife PSA might identify men at high risk while minimizing screening in those men at low-risk. |
| Kovac (2020) [21] | NCCN | Secondary analysis of randomised trial (PLCO) | Competing risk regression to model risk of prostate cancer | Single test | Prostate cancer incidence, clinically significant prostate cancer | No | findings suggest that repeated screening can be less frequent among men aged 55 to 60 years with a low baseline PSA level (<2 ng/mL) and possibly discontinued among those with baseline PSA levels of less than 1 ng/mL. |
| Ulmert (2008) [22] | NCCN | Retrospective case control | Logistic regression to determine association between PSA at age 50 and risk of prostate cancer | Single test | Advanced prostate cancer diagnosis | No | Suggested the possibility of using an early PSA test to risk-stratify patients so that patients at highest risk receive intensive screening efforts. |
| Smith (2018) [23] | American Cancer Society | Review of guidelines | Summarises the current ACS cancer screening guidelines, including current recommendations, updates, and guidance related to early cancer detection when a direct recommendation for screening cannot be made | NA | NA | No | For PSA levels less than 2.5 ng/mL, screening intervals can be extended to every 2 years, and screening should be conducted annually for patients with PSA levels of 2.5 ng/mL or higher |
| Wolf (2010) [24] | American Cancer Society | Guideline | NA | NA | NA | No | For men whose PSA is less than 2.5 ng/mL, screening intervals can be extended to every 2 years. Men with higher PSA values should be tested annually. |
| Kipelainen (2013) [25] | Cancer Australia | Secondary analysis of randomised trial (ERSPC) | HR Cox model | Multiple tests | Incidence, mortality prostate cancer | No | No interval recommendation. Conservative screening protocol at 12 years of follow-up, resulted in a small, non-statistically significant, mortality reduction |
| Kjellman (2009) [26] | Cancer Australia | Randomised trial | Estimated the prostate specific mortality rates as well as all cause mortality and calculated IRR using Poisson regression | Single test | Prostate specific mortality | No | No interval recommendation. Found no effect of the screening procedure on the risk of death from prostate cancer and other causes of death |
| Bokhorst (2014) [27] | Cancer Australia | Secondary analysis randomised trial (ERSPC) | Prostate cancer mortality | Multiple | Mortality prostate cancer | No | No interval recommendation.4 year interval screening reduced the risk of dying from prostate cancer up to 51% |
| Hugosson (2010) [28] | Cancer Australia | Secondary analysis randomised trial (ERSPC ) | Risk of dying prostate cancer | Multiple | Prostate cancer specific mortality | No | No interval recommendation |
| Labrie (2004) [29] | Cancer Australia | Randomised trial (Quebec study) | Cox regression | Multiple | Prostate cancer specific mortality | No | Recommend annual testing |
| Roobol (2013) [30] | Cancer Australia | Randomised trial (ERSPC) | Prostate cancer specific mortality analyses using Poisson regression | Multiple | Prostate cancer specific mortality | No | 4-year interval. Systematic PSA-based screening reduced prostate cancer specific mortality by 32% in the age range of 55-69 yr. |
| Sandblom (2004)[31] | Cancer Australia | Randomised trial |  | Multiple | Prostate cancer incidence | No | 3 year interval |
| Andriole (2009) [32] | Cancer Australia | Randomised trial (PLCO) | [33] | Multiple | Prostate-cancer mortality | No | No recommended interval. Annual screening found no difference in mortality |
| Sandblom (2011) [34] | Cancer Australia | Randomised trial | Cox risk of death | Multiple | Prostate cancer specific mortality | No | No recommended interval. Three year screening found no difference in mortality |
| Basch (2012) [35] | New Zealand | Guideline | Clinical opinion | NA | NA | No | No recommended interval. Recommended for shared decision making |
| Catalona (2011) [36] | New Zealand | Prospective study | Diagnostic accuracy of the prostate health index (phi) | Single | Prostate cancer diagnosis (gleason ≥7) | No | No recommended interval. Phi may be useful to reduce biopsies in men age ≥50 years with PSA 2–10 ng/mL and negative DRE |
| DeSantis (2019) [37] | South Africa | Review of reports | Review of Cancer Audit and SEER data | NA | Prostate cancer diagnosis | No | No recommended interval |
| Hugosson (2019) [38] | France | Secondary analysis randomised trial (ERSPC) | Rate ratio, number needed to diagnose and the number needed to be invited for screening to prevent one death | NA | Prostate cancer specific mortality | No | No recommended interval. PSA screening reduces prostate specific mortality. Repeated screening may be important to reduce prostate cancer mortality on a population level. |

References for Supplementary Table 1

1. Vickers, A.J., et al., *Strategy for detection of prostate cancer based on relation between prostate specific antigen at age 40-55 and long term risk of metastasis: case-control study.* BMJ, 2013. **346**: p. f2023.

2. Carlsson, S., et al., *Influence of blood prostate specific antigen levels at age 60 on benefits and harms of prostate cancer screening: population based cohort study.* Bmj, 2014. **348**: p. g2296.

3. Gelfond, J., et al., *Intermediate-Term Risk of Prostate Cancer is Directly Related to Baseline Prostate Specific Antigen: Implications for Reducing the Burden of Prostate Specific Antigen Screening.* J Urol, 2015. **194**(1): p. 46-51.

4. Roobol, M.J., D.W. Roobol, and F.H. Schroder, *Is additional testing necessary in men with prostate-specific antigen levels of 1.0 ng/mL or less in a population-based screening setting? (ERSPC, section Rotterdam).* Urology, 2005. **65**(2): p. 343-6.

5. Preston, M.A., et al., *Baseline Prostate-Specific Antigen Levels in Midlife Predict Lethal Prostate Cancer.* J Clin Oncol, 2016. **34**(23): p. 2705-11.

6. Andriole, G.L., et al., *Prostate cancer screening in the randomized Prostate, Lung, Colorectal, and Ovarian Cancer Screening Trial: mortality results after 13 years of follow-up.* J Natl Cancer Inst, 2012. **104**(2): p. 125-32.

7. Vickers, A.J., et al., *Prostate specific antigen concentration at age 60 and death or metastasis from prostate cancer: case-control study.* Bmj, 2010. **341**: p. c4521.

8. Heijnsdijk, E.A.M., et al., *Lifetime Benefits and Harms of Prostate-Specific Antigen-Based Risk-Stratified Screening for Prostate Cancer.* J Natl Cancer Inst, 2020. **112**(10): p. 1013-1020.

9. Schröder, F.H., et al., *Screening and prostate cancer mortality: results of the European Randomised Study of Screening for Prostate Cancer (ERSPC) at 13 years of follow-up.* Lancet, 2014. **384**(9959): p. 2027-35.

10. Schröder, F.H., et al., *Prostate-cancer mortality at 11 years of follow-up.* N Engl J Med, 2012. **366**(11): p. 981-90.

11. Gulati, R., J.L. Gore, and R. Etzioni, *Comparative effectiveness of alternative prostate-specific antigen--based prostate cancer screening strategies: model estimates of potential benefits and harms.* Ann Intern Med, 2013. **158**(3): p. 145-53.

12. Heijnsdijk, E.A., et al., *Quality-of-life effects of prostate-specific antigen screening.* N Engl J Med, 2012. **367**(7): p. 595-605.

13. Ross, K.S., et al., *Comparative efficiency of prostate-specific antigen screening strategies for prostate cancer detection.* JAMA, 2000. **284**(11): p. 1399-405.

14. Thompson, I.M., et al., *Assessing prostate cancer risk: results from the Prostate Cancer Prevention Trial.* Journal of the National Cancer Institute, 2006. **98 8**: p. 529-34.

15. Lilja, H., et al., *Long-term prediction of prostate cancer up to 25 years before diagnosis of prostate cancer using prostate kallikreins measured at age 44 to 50 years.* J Clin Oncol, 2007. **25**(4): p. 431-6.

16. Loeb, S., S. Carlsson, and R.S. Braithwaite, *Prostate cancer: modeling the outcomes of prostate cancer screening.* Nature Reviews Urology, 2012. **9**(4): p. 183-5.

17. Eastham, J.A., et al., *Variation of serum prostate-specific antigen levels: an evaluation of year-to-year fluctuations.* Jama, 2003. **289**(20): p. 2695-700.

18. van Leeuwen, P.J., et al., *Balancing the harms and benefits of early detection of prostate cancer.* Cancer, 2010. **116**(20): p. 4857-65.

19. Vertosick, E.A., et al., *Prespecified 4-Kallikrein Marker Model at Age 50 or 60 for Early Detection of Lethal Prostate Cancer in a Large Population Based Cohort of Asymptomatic Men Followed for 20 Years.* J Urol, 2020. **204**(2): p. 281-288.

20. Preston, M.A., et al., *Baseline Prostate-specific Antigen Level in Midlife and Aggressive Prostate Cancer in Black Men.* Eur Urol, 2019. **75**(3): p. 399-407.

21. Kovac, E., et al., *Association of Baseline Prostate-Specific Antigen Level With Long-term Diagnosis of Clinically Significant Prostate Cancer Among Patients Aged 55 to 60 Years: A Secondary Analysis of a Cohort in the Prostate, Lung, Colorectal, and Ovarian (PLCO) Cancer Screening Trial.* JAMA Netw Open, 2020. **3**(1): p. e1919284.

22. Ulmert, D., et al., *Prostate-specific antigen at or before age 50 as a predictor of advanced prostate cancer diagnosed up to 25 years later: a case-control study.* BMC Med, 2008. **6**: p. 6.

23. Smith, R.A., et al., *Cancer screening in the United States, 2018: A review of current American Cancer Society guidelines and current issues in cancer screening.* CA Cancer J Clin, 2018. **68**(4): p. 297-316.

24. Wolf, A.M., et al., *American Cancer Society guideline for the early detection of prostate cancer: update 2010.* CA Cancer J Clin, 2010. **60**(2): p. 70-98.

25. Kilpeläinen, T.P., et al., *Prostate cancer mortality in the Finnish randomized screening trial.* J Natl Cancer Inst, 2013. **105**(10): p. 719-25.

26. Kjellman, A., et al., *15-year followup of a population based prostate cancer screening study.* J Urol, 2009. **181**(4): p. 1615-21; discussion 1621.

27. Bokhorst, L.P., et al., *Prostate-specific antigen-based prostate cancer screening: reduction of prostate cancer mortality after correction for nonattendance and contamination in the Rotterdam section of the European Randomized Study of Screening for Prostate Cancer.* Eur Urol, 2014. **65**(2): p. 329-36.

28. Hugosson, J., et al., *Mortality results from the Göteborg randomised population-based prostate-cancer screening trial.* Lancet Oncol, 2010. **11**(8): p. 725-32.

29. Labrie, F., et al., *Screening decreases prostate cancer mortality: 11-year follow-up of the 1988 Quebec prospective randomized controlled trial.* Prostate, 2004. **59**(3): p. 311-8.

30. Roobol, M.J., et al., *Screening for prostate cancer: results of the Rotterdam section of the European randomized study of screening for prostate cancer.* Eur Urol, 2013. **64**(4): p. 530-9.

31. Sandblom, G., et al., *Clinical consequences of screening for prostate cancer: 15 years follow-up of a randomised controlled trial in Sweden.* Eur Urol, 2004. **46**(6): p. 717-23; discussion 724.

32. Andriole, G.L., et al., *Mortality results from a randomized prostate-cancer screening trial.* N Engl J Med, 2009. **360**(13): p. 1310-9.

33. Parker, C., et al., *Prostate cancer: ESMO Clinical Practice Guidelines for diagnosis, treatment and follow-up<sup>&#x2020;</sup>.* Annals of Oncology, 2020. **31**(9): p. 1119-1134.

34. Sandblom, G., et al., *Randomised prostate cancer screening trial: 20 year follow-up.* Bmj, 2011. **342**: p. d1539.

35. Basch, E., et al., *Screening for prostate cancer with prostate-specific antigen testing: American Society of Clinical Oncology Provisional Clinical Opinion.* J Clin Oncol, 2012. **30**(24): p. 3020-5.

36. Catalona, W.J., et al., *A multicenter study of [-2]pro-prostate specific antigen combined with prostate specific antigen and free prostate specific antigen for prostate cancer detection in the 2.0 to 10.0 ng/ml prostate specific antigen range.* J Urol, 2011. **185**(5): p. 1650-5.

37. DeSantis, C.E., et al., *Cancer statistics for African Americans, 2019.* CA: A Cancer Journal for Clinicians, 2019. **69**(3): p. 211-233.

38. Hugosson, J., et al., *A 16-yr Follow-up of the European Randomized study of Screening for Prostate Cancer.* Eur Urol, 2019. **76**(1): p. 43-51.

## Supplementary Table 2: Individual AGREE II reviewer scores by guideline (pages 39-52)

Table 2.1: EAU - EANM - ESTRO - ESUR - ISUP – SIOG (2024)

Table 2.2: American Urological Association (2023)

Table 2.3: Memorial Sloan Kettering (2016)

## Table 2.4: National Comprehensive Cancer Network (NCCN) (2023)

## Table 2.5: Sociedad Espanola de Oncologia Medica (SEOM) (2014)

## Table 2.6: Canadian Urological Association (2022)

## Table 2.7: Australia Cancer Council (2016)

## Table 2.8: Prostate Cancer Working Group and Ministry of Health (2015)

## Table 2.9: American Cancer Society (2023)

## Table 2.10: South Africa Urological Association (2024)

## Table 2.11: French Urological Association (2022)

## Table 2: Individual AGREE II reviewer scores by Guideline

## Table 2.1 EAU - EANM - ESTRO - ESUR - ISUP – SIOG (2024)

| AGREE II Question | Score Reviewer 1 | Score Reviewer 2 |
| --- | --- | --- |
| 1 objectives | 5 | 6 |
| 2 questions | 2 | 4 |
| 3 population | 3 | 6 |
| 4 group membership | 6 | 5 |
| 5 target population and preferences PPI | 5 | 5 |
| 6 target users | 6 | 7 |
| 7 search methods | 7 | 7 |
| 8 evidence and selection | 5 | 7 |
| 9 strengths and limitations | 6 | 7 |
| 10 formulation of recommendations | 6 | 6 |
| 11 consideration of benefits and harms | 4 | 7 |
| 12 link between recommendations and evidence | 5 | 6 |
| 13 external review | 2 | 3 |
| 14 updating procedure | 2 | 4 |
| 15 specific and unambiguous recommendations | 6 | 5 |
| 16 management options | 6 | 6 |
| 17 identifiable key recommendations | 6 | 5 |
| 18 facilitators and barriers to application | 5 | 2 |
| 19 implementation advice/tools | 3 | 2 |
| 20 resource implications | 3 | 5 |
| 21 monitoring/auditing criteria | 6 | 2 |
| 22 funding body | 7 | 7 |
| 23 competing interests | 7 | 7 |

## Table 2.2: American Urological Association (2023)

| AGREE II Question | Score Reviewer 1 | Score Reviewer 2 |
| --- | --- | --- |
| 1 objectives | 6 | 6 |
| 2 questions | 7 | 6 |
| 3 population | 7 | 7 |
| 4 group membership | 7 | 7 |
| 5 target population and preferences PPI | 7 | 4 |
| 6 target users | 6 | 6 |
| 7 search methods | 7 | 7 |
| 8 evidence and selection | 7 | 7 |
| 9 strengths and limitations | 6 | 6 |
| 10 formulation of recommendations | 6 | 6 |
| 11 consideration of benefits and harms | 6 | 7 |
| 12 link between recommendations and evidence | 6 | 7 |
| 13 external review | 7 | 7 |
| 14 updating procedure | 2 | 2 |
| 15 specific and unambiguous recommendations | 5 | 5 |
| 16 management options | 4 | 3 |
| 17 identifiable key recommendations | 6 | 6 |
| 18 facilitators and barriers to application | 5 | 4 |
| 19 implementation advice/tools | 4 | 3 |
| 20 resource implications | 4 | 3 |
| 21 monitoring/auditing criteria | 6 | 3 |
| 22 funding body | 5 | 7 |
| 23 competing interests | 7 | 7 |

## Table 2.3: Memorial Sloan Kettering (2016)

| AGREE II Question | Score Reviewer 1 | Score Reviewer 2 |
| --- | --- | --- |
| 1 objectives | 6 | 7 |
| 2 questions | 6 | 5 |
| 3 population | 7 | 7 |
| 4 group membership | 1 | 3 |
| 5 target population and preferences PPI | 1 | 3 |
| 6 target users | 7 | 5 |
| 7 search methods | 1 | 1 |
| 8 evidence and selection | 1 | 4 |
| 9 strengths and limitations | 2 | 3 |
| 10 formulation of recommendations | 5 | 5 |
| 11 consideration of benefits and harms | 5 | 7 |
| 12 link between recommendations and evidence | 5 | 6 |
| 13 external review | 2 | 5 |
| 14 updating procedure | 1 | 1 |
| 15 specific and unambiguous recommendations | 6 | 7 |
| 16 management options | 2 | 3 |
| 17 identifiable key recommendations | 6 | 7 |
| 18 facilitators and barriers to application | 2 | 2 |
| 19 implementation advice/tools | 2 | 4 |
| 20 resource implications | 4 | 2 |
| 21 monitoring/auditing criteria | 5 | 2 |
| 22 funding body | 7 | 7 |
| 23 competing interests | 7 | 7 |

## Table 2.4: National Cancer Comprehensive Network (NCCN) (2023)

| AGREE II Question | Score Reviewer 1 | Score Reviewer 2 |
| --- | --- | --- |
| 1 objectives | 6 | 6 |
| 2 questions | 5 | 5 |
| 3 population | 5 | 6 |
| 4 group membership | 5 | 7 |
| 5 target population and preferences PPI | 2 | 2 |
| 6 target users | 7 | 7 |
| 7 search methods | 3 | 2 |
| 8 evidence and selection | 3 | 2 |
| 9 strengths and limitations | 2 | 4 |
| 10 formulation of recommendations | 4 | 5 |
| 11 consideration of benefits and harms | 5 | 4 |
| 12 link between recommendations and evidence | 4 | 7 |
| 13 external review | 2 | 2 |
| 14 updating procedure | 1 | 2 |
| 15 specific and unambiguous recommendations | 6 | 5 |
| 16 management options | 6 | 4 |
| 17 identifiable key recommendations | 6 | 5 |
| 18 facilitators and barriers to application | 4 | 2 |
| 19 implementation advice/tools | 4 | 4 |
| 20 resource implications | 4 | 2 |
| 21 monitoring/auditing criteria | 4 | 2 |
| 22 funding body | 3 | 7 |
| 23 competing interests | 6 | 7 |

## Table 2.5: Sociedad Espanola de Oncologia Medica (SEOM) (2014)

| AGREE II Question | Score Reviewer 1 | Score Reviewer 2 |
| --- | --- | --- |
| 1 objectives | 4 | 5 |
| 2 questions | 4 | 4 |
| 3 population | 4 | 4 |
| 4 group membership | 2 | 5 |
| 5 target population and preferences PPI | 1 | 2 |
| 6 target users | 4 | 2 |
| 7 search methods | 1 | 1 |
| 8 evidence and selection | 1 | 1 |
| 9 strengths and limitations | 5 | 4 |
| 10 formulation of recommendations | 5 | 4 |
| 11 consideration of benefits and harms | 5 | 4 |
| 12 link between recommendations and evidence | 5 | 2 |
| 13 external review | 2 | 1 |
| 14 updating procedure | 1 | 1 |
| 15 specific and unambiguous recommendations | 6 | 6 |
| 16 management options | 2 | 5 |
| 17 identifiable key recommendations | 6 | 6 |
| 18 facilitators and barriers to application | 4 | 2 |
| 19 implementation advice/tools | 4 | 2 |
| 20 resource implications | 4 | 2 |
| 21 monitoring/auditing criteria | 3 | 2 |
| 22 funding body | 1 | 1 |
| 23 competing interests | 7 | 7 |

## Table 2.6: Canadian Urological Association (2022)

| AGREE II Question | Score Reviewer 1 | Score Reviewer 2 |
| --- | --- | --- |
| 1 objectives | 6 | 7 |
| 2 questions | 7 | 7 |
| 3 population | 6 | 6 |
| 4 group membership | 3 | 6 |
| 5 target population and preferences PPI | 2 | 5 |
| 6 target users | 5 | 5 |
| 7 search methods | 7 | 7 |
| 8 evidence and selection | 7 | 7 |
| 9 strengths and limitations | 6 | 6 |
| 10 formulation of recommendations | 6 | 6 |
| 11 consideration of benefits and harms | 6 | 6 |
| 12 link between recommendations and evidence | 6 | 7 |
| 13 external review | 4 | 4 |
| 14 updating procedure | 2 | 1 |
| 15 specific and unambiguous recommendations | 6 | 7 |
| 16 management options | 4 | 5 |
| 17 identifiable key recommendations | 6 | 7 |
| 18 facilitators and barriers to application | 4 | 5 |
| 19 implementation advice/tools | 4 | 5 |
| 20 resource implications | 5 | 4 |
| 21 monitoring/auditing criteria | 5 | 2 |
| 22 funding body | 1 | 1 |
| 23 competing interests | 7 | 7 |

## Table 2.7: Cancer Council Australia (2016)

| AGREE II Question | Score Reviewer 1 | Score Reviewer 2 |
| --- | --- | --- |
| 1 objectives | 7 | 6 |
| 2 questions | 7 | 6 |
| 3 population | 7 | 6 |
| 4 group membership | 7 | 7 |
| 5 target population and preferences PPI | 7 | 4 |
| 6 target users | 7 | 6 |
| 7 search methods | 7 | 7 |
| 8 evidence and selection | 7 | 6 |
| 9 strengths and limitations | 6 | 6 |
| 10 formulation of recommendations | 6 | 3 |
| 11 consideration of benefits and harms | 6 | 7 |
| 12 link between recommendations and evidence | 6 | 7 |
| 13 external review | 4 | 4 |
| 14 updating procedure | 7 | 7 |
| 15 specific and unambiguous recommendations | 6 | 6 |
| 16 management options | 5 | 6 |
| 17 identifiable key recommendations | 6 | 7 |
| 18 facilitators and barriers to application | 7 | 7 |
| 19 implementation advice/tools | 7 | 7 |
| 20 resource implications | 6 | 7 |
| 21 monitoring/auditing criteria | 5 | 3 |
| 22 funding body | 6 | 7 |
| 23 competing interests | 7 | 7 |

## Table 2.8: American Cancer Society (2023)

| AGREE II Question | Score Reviewer 1 | Score Reviewer 2 |
| --- | --- | --- |
| 1 objectives | 5 | 5 |
| 2 questions | 6 | 4 |
| 3 population | 6 | 5 |
| 4 group membership | 6 | 5 |
| 5 target population and preferences PPI | 6 | 4 |
| 6 target users | 6 | 5 |
| 7 search methods | 4 | 2 |
| 8 evidence and selection | 4 | 2 |
| 9 strengths and limitations | 4 | 2 |
| 10 formulation of recommendations | 3 | 2 |
| 11 consideration of benefits and harms | 5 | 3 |
| 12 link between recommendations and evidence | 3 | 2 |
| 13 external review | 5 | 2 |
| 14 updating procedure | 1 | 2 |
| 15 specific and unambiguous recommendations | 6 | 6 |
| 16 management options | 5 | 4 |
| 17 identifiable key recommendations | 6 | 5 |
| 18 facilitators and barriers to application | 4 | 3 |
| 19 implementation advice/tools | 4 | 3 |
| 20 resource implications | 5 | 3 |
| 21 monitoring/auditing criteria | 5 | 3 |
| 22 funding body | 1 | 1 |
| 23 competing interests | 1 | 1 |

## Table 2.9: Prostate Cancer Working Group and Ministry of Health (2015)

| AGREE II Question | Score Reviewer 1 | Score Reviewer 2 |
| --- | --- | --- |
| 1 objectives | 5 | 6 |
| 2 questions | 2 | 5 |
| 3 population | 5 | 5 |
| 4 group membership | 5 | 2 |
| 5 target population and preferences PPI | 2 | 3 |
| 6 target users | 5 | 4 |
| 7 search methods | 1 | 1 |
| 8 evidence and selection | 1 | 3 |
| 9 strengths and limitations | 2 | 5 |
| 10 formulation of recommendations | 2 | 4 |
| 11 consideration of benefits and harms | 6 | 4 |
| 12 link between recommendations and evidence | 6 | 4 |
| 13 external review | 6 | 5 |
| 14 updating procedure | 1 | 1 |
| 15 specific and unambiguous recommendations | 6 | 4 |
| 16 management options | 6 | 5 |
| 17 identifiable key recommendations | 6 | 7 |
| 18 facilitators and barriers to application | 3 | 4 |
| 19 implementation advice/tools | 5 | 6 |
| 20 resource implications | 4 | 2 |
| 21 monitoring/auditing criteria | 5 | 2 |
| 22 funding body | 4 | 1 |
| 23 competing interests | 2 | 1 |

## Table 2.10 South Africa Urological Association (2024)

| AGREE II Question | Score Reviewer 1 | Score Reviewer 2 |
| --- | --- | --- |
| 1 objectives | 5 | 4 |
| 2 questions | 4 | 4 |
| 3 population | 5 | 6 |
| 4 group membership | 4 | 2 |
| 5 target population and preferences PPI | 3 | 2 |
| 6 target users | 4 | 3 |
| 7 search methods | 2 | 2 |
| 8 evidence and selection | 1 | 2 |
| 9 strengths and limitations | 2 | 2 |
| 10 formulation of recommendations | 2 | 2 |
| 11 consideration of benefits and harms | 5 | 3 |
| 12 link between recommendations and evidence | 6 | 6 |
| 13 external review | 4 | 5 |
| 14 updating procedure | 1 | 1 |
| 15 specific and unambiguous recommendations | 5 | 5 |
| 16 management options | 5 | 5 |
| 17 identifiable key recommendations | 5 | 2 |
| 18 facilitators and barriers to application | 3 | 2 |
| 19 implementation advice/tools | 2 | 2 |
| 20 resource implications | 3 | 2 |
| 21 monitoring/auditing criteria | 1 | 2 |
| 22 funding body | 3 | 1 |
| 23 competing interests | 1 | 1 |

## Table 2.11: France Urological Association (2022)

| AGREE II Question | Score Reviewer 1 | Score Reviewer 2 |
| --- | --- | --- |
| 1 objectives | 5 | 3 |
| 2 questions | 4 | 5 |
| 3 population | 3 | 3 |
| 4 group membership | 3 | 2 |
| 5 target population and preferences PPI | 2 | 2 |
| 6 target users | 3 | 4 |
| 7 search methods | 5 | 6 |
| 8 evidence and selection | 2 | 2 |
| 9 strengths and limitations | 3 | 2 |
| 10 formulation of recommendations | 4 | 4 |
| 11 consideration of benefits and harms | 5 | 5 |
| 12 link between recommendations and evidence | 4 | 3 |
| 13 external review | 6 | 6 |
| 14 updating procedure | 1 | 2 |
| 15 specific and unambiguous recommendations | 6 | 6 |
| 16 management options | 6 | 5 |
| 17 identifiable key recommendations | 5 | 5 |
| 18 facilitators and barriers to application | 4 | 3 |
| 19 implementation advice/tools | 4 | 3 |
| 20 resource implications | 4 | 3 |
| 21 monitoring/auditing criteria | 2 | 3 |
| 22 funding body | 2 | 3 |
| 23 competing interests | 7 | 7 |
